# Using Artificial Intelligence to Assess the Teratogenic Risk of Vitamin A Supplements

**DOI:** 10.7759/cureus.45931

**Published:** 2023-09-25

**Authors:** Swathi Holla, Dina H Zamil, Praneet S Paidisetty, Leonard K Wang, Rajani Katta

**Affiliations:** 1 Dermatology, Baylor College of Medicine, Houston, USA; 2 Dermatology, McGovern Medical School, The University of Texas Health Science Center, Houston, USA; 3 Dermatology, John Sealy School of Medicine, The University of Texas Medical Branch, Galveston, USA

**Keywords:** isotretinoin, vitamin a supplements, acne, ethics, teratogenic medications, ai & robotics in healthcare

## Abstract

Vitamin A in high doses has been found to be highly teratogenic, leading to severe fetal abnormalities if exposure occurs during pregnancy. Hence, prescription vitamin A acne medications like isotretinoin are highly regulated via programs such as iPledge, which intend to avert fetal exposure to isotretinoin and to educate healthcare providers, pharmacists, and patients about the significant risks associated with isotretinoin and its appropriate usage conditions. However, over-the-counter (OTC) vitamin A supplements are not subject to these requirements, and calculating the vitamin A content of these supplements can be difficult due to the lack of Food and Drug Administration (FDA) regulations and inconsistencies in labeling. If the necessary information is provided, ChatGPT, a generative artificial intelligence (AI) tool, can help the general public calculate the vitamin A content of supplements. Nonetheless, supplement manufacturers do not always provide the data necessary for these calculations.

## Editorial

Vitamin A and its derivatives are commonly used in over-the-counter (OTC) supplements for dermatological conditions, including acne [1,2]. However, consuming vitamin A in high doses has been found to have teratogenic effects and is strongly associated with neural tube defects [3]. As with all supplements, OTC vitamin A supplements are not subject to rigorous Food and Drug Administration (FDA) regulations [1]. Thus, inconsistencies in the reporting of vitamin A type and units of measurement on product labels can make it difficult to determine the vitamin A content in these supplements. This information is essential to determine whether the vitamin A dosage of OTC supplements is teratogenic [1].

The rate of unplanned pregnancies in women taking prescription isotretinoin, a high-dose vitamin A derivative, has declined since the implementation of iPledge, a program that provides users with mandatory precautions to prevent pregnancy, including a requirement for monthly pregnancy tests and birth control [4]. However, iPledge monitoring does not extend to women taking OTC vitamin A supplements, and hence there is a greater risk of fetal complications among them. This is especially pertinent given recent restrictions on abortion access across much of the United States [5]. It is crucial that women of reproductive age understand the potential adverse effects of the medications and supplements they consume.

Calculating whether a vitamin A supplement exceeds the teratogenic threshold can be complicated, even for a physician. The FDA requires that manufacturers provide the measurement of vitamin A levels in micrograms of retinol activity equivalents (mcg RAE) on the labels, while studies of vitamin A have reported the teratogenic threshold in International Units (IUs) [1]. When the subtype of vitamin A (retinol and retinyl esters versus beta-carotene) and quantitative content in mcg RAE are available, the content in IUs can be determined using a conversion factor. In other words, 1 mcg RAE in retinol or retinyl esters is equivalent to 3.33 IUs, and 1 mcg RAE beta-carotene is equivalent to 3.33 or 20 IUs, depending on the source (supplemental or dietary beta-carotene). Once the vitamin A content in mcg RAE is converted to IUs, one can determine whether the vitamin A level exceeds the recommended gestational levels (approximately 2,565 IUs) as well as the teratogenic threshold (10,000 IUs) [1].

Furthermore, these calculations can be challenging for the general public who may be unaware of concepts such as the teratogenic threshold, various subtypes of vitamin A, or the various units to measure vitamin A dosage. In light of this, we tried to determine whether artificial intelligence (AI) may be useful to make these calculations easier and more accessible. We conversed with ChatGPT 3.5, a generative AI tool, to determine what information it could provide users to calculate a supplement’s vitamin A content in IUs, and whether it could determine if the calculated content exceeded the teratogenic threshold of 10,000 IUs. We asked ChatGPT 3.5 to calculate vitamin A content in five specific vitamin A supplements, for links to purchase the supplements, for nutrition labels, and for values from nutrition labels. We also asked ChatGPT 3.5 to advise if specific vitamin A supplements were teratogenic.

Notably, we found that the AI tool requires the same information that a person would utilize to calculate vitamin A levels in IUs and assess for the risk of teratogenicity. These requirements include vitamin A type and vitamin A dosage in mcg RAE. When these data were input into the tool, ChatGPT was able to accurately calculate vitamin A levels for all five supplements and explain the process for determining vitamin A content in IUs. ChatGPT 3.5 was also able to correctly determine whether vitamin A content exceeded the teratogenic threshold and provided suggestions to visit a healthcare provider for users who may have concerns about supplement levels. The limitations of the AI tool include a knowledge base limited to before September 2021 and an inability to read images, such as Supplement Facts labels, and to actively browse and gather data from websites. Although GPT-4 may have addressed these restrictions, this program is a paid subscription service with restricted access. Given our study solely used GPT-3.5, our findings are limited to answers provided by GPT-3.5. However, GPT-3.5 is a more accessible multimodal large language model for the general public compared to GPT-4. Future research should assess the capabilities of GPT-4 and subsequent versions of ChatGPT, as well as other AI tools. Larger-scale studies that strive to collect more empirical data could further evaluate AI capabilities to assess supplements.

ChatGPT can be a powerful adjunct for the analysis of the vitamin A content of OTC supplements. It can increase efficiency for calculations that are otherwise arduous and time-consuming when it is given the appropriate information. However, previous studies have found that manufacturers often do not display the two elements (vitamin A subtype and vitamin A content in mcg RAE) that ChatGPT needs to perform these calculations [1]. Additionally, the use of ChatGPT for this purpose is contingent on users being aware of the adverse effects of high-dose vitamin A. Many OTC vitamin A users simply may not know the dangers associated with vitamin A and OTC supplements. Finally, the use of ChatGPT 3.5 for supplement risk assessment raises certain ethical questions. ChatGPT is not a substitute for professional medical advice and has not been studied or FDA-approved for medical-related calculations or decision-making. Thus, we recommend that the FDA continues to require manufacturers to disclose pertinent information such as the relative proportions of vitamin A subtypes in percentages and levels in mcg RAE to enable calculation and risk assessment [1]. Overall, although generative chatbots can help simplify complex medical knowledge, with regard to vitamin A supplements, they cannot replace proper counseling but instead may be used to bridge the knowledge gap between clinicians and the public.
